# Supplementary material for: Identifying HIV-1 Transmission Clusters in Uzbekistan through Analysis of Molecular Surveillance Data
Source: Viruses. 2022 Jul 29;14(8):1675. doi: 10.3390/v14081675 (PMC9413238; doi:10.3390/v14081675)
Supplement: Supplementary file 1 [file viruses-14-01675-s001.zip › Supplementary Files/Table S1.pdf]

Figure S1. General characteristics of HIV-1 infected patients from Uzbekistan stratified by subtypes.

Characteristic [1]	Subtype CRF02_AG, *N* (%)	Sub-subtype A6, *N* (%)	*P* value [2]
**Age (years)**			0.114
<25	29 (15.4)	9 (8.0)	
25-35	63 (33.3)	32 (28.3)	
35-45	69 (36.5)	54 (47.7)	
>45	28 (14.8)	18 (16.0)	
**Gender**			0.057
Male	89 (47.1)	67 (59.3)	
Female	100 (52.9)	46 (40.7)	
**Transmission category**			0.084
IDU	61 (32.3)	35 (31.0)	
HET	66 (34.9)	51 (45.1)	
UNK	62 (32.8)	27 (23.9)	
**Region in Uzbekistan [3]**			<0.001
Andijan*	45 (23.8)	9 (8.0)	
Bukhara	1 (0.5)	5 (4.4)	
Fergana*	16 (8.5)	7 (6.2)	
Jizzakh	1 (0.5)	4 (3.5)	
Karakalpakstan	8 (4.2)	0	
Xorazm	1 (0.5)	7 (6.2)	
Namangan*	6 (3.2)	0	
Navoiy	1 (0.5)	3 (2.7)	
Qashqadaryo	6 (3.2)	15 (13.3)	
Samarkand	16 (8.5)	23 (20.3)	
Sirdaryo	14 (7.4)	5 (4.4)	
Surxondaryo	7 (3.7)	3 (2.7)	
Tashkent*	67 (35.5)	32 (28.3)	
**Total**	189 (100.0)	113 (100.0)	

[1] Characteristic indicated only for participants in the Nationwide Surveillance of HIV drug resistance (NS-HIVDR; see Materials and Methods); at the end of sampling in 2016, 36,000 people living with HIV were registered in Uzbekistan, including 12,000 women (15+) and 20,000 men (15+). [2] Value for the difference between HIV-1 subtypes (Pearson's chi-squared and/or Fisher's exact test). [3] Regions marked with an asterisk were considered as a single group under the name of North Eastern Region (NER) for some types of analysis (see main text).
Abbreviations: HET, heterosexual contacts; IDU, injection drug users; UNK, unknown.
